# Resource partitioning facilitates coexistence in sympatric cetaceans in the California Current

**DOI:** 10.1002/ece3.3409

**Published:** 2017-09-27

**Authors:** Sabrina Fossette, Briana Abrahms, Elliott L. Hazen, Steven J. Bograd, Kelly M. Zilliacus, John Calambokidis, Julia A. Burrows, Jeremy A. Goldbogen, James T. Harvey, Baldo Marinovic, Bernie Tershy, Donald A. Croll

**Affiliations:** ^1^ Environmental Research Division NOAA Southwest Fisheries Science Center Monterey CA USA; ^2^ Department of Ecology and Evolutionary Biology University of California Santa Cruz Santa Cruz CA USA; ^3^ Cascadia Research Collective Olympia WA USA; ^4^ Division of Marine Science and Conservation Duke University Marine Laboratory Beaufort NC USA; ^5^ Moss Landing Marine Laboratories Moss Landing CA USA; ^6^ Department of Biology Hopkins Marine Station Stanford University Pacific Grove CA USA; ^7^ Present address: Department of Biodiversity, Conservation and Attractions 17 Dick Perry Av Kensington WA 6151 Australia

**Keywords:** *Balaenoptera*, foraging ecology, interspecific competition, resource partitioning, species coexistence, trophic position

## Abstract

Resource partitioning is an important process driving habitat use and foraging strategies in sympatric species that potentially compete. Differences in foraging behavior are hypothesized to contribute to species coexistence by facilitating resource partitioning, but little is known on the multiple mechanisms for partitioning that may occur simultaneously. Studies are further limited in the marine environment, where the spatial and temporal distribution of resources is highly dynamic and subsequently difficult to quantify. We investigated potential pathways by which foraging behavior may facilitate resource partitioning in two of the largest co‐occurring and closely related species on Earth, blue (*Balaenoptera musculus*) and humpback (*Megaptera novaeangliae*) whales. We integrated multiple long‐term datasets (line‐transect surveys, whale‐watching records, net sampling, stable isotope analysis, and remote‐sensing of oceanographic parameters) to compare the diet, phenology, and distribution of the two species during their foraging periods in the highly productive waters of Monterey Bay, California, USA within the California Current Ecosystem. Our long‐term study reveals that blue and humpback whales likely facilitate sympatry by partitioning their foraging along three axes: trophic, temporal, and spatial. Blue whales were specialists foraging on krill, predictably targeting a seasonal peak in krill abundance, were present in the bay for an average of 4.7 months, and were spatially restricted at the continental shelf break. In contrast, humpback whales were generalists apparently feeding on a mixed diet of krill and fishes depending on relative abundances, were present in the bay for a more extended period (average of 6.6 months), and had a broader spatial distribution at the shelf break and inshore. Ultimately, competition for common resources can lead to behavioral, morphological, and physiological character displacement between sympatric species. Understanding the mechanisms for species coexistence is both fundamental to maintaining biodiverse ecosystems, and provides insight into the evolutionary drivers of morphological differences in closely related species.

## 1 INTRODUCTION

Understanding the role of resource competition and partitioning between sympatric species in driving differential habitat use and foraging strategies has long been a fundamental question in ecology (Brown & Wilson, 1956; Fenchel, 1975; Grant, 1972). Resource partitioning promotes the coexistence of species that compete for shared limited resources (Schoener, 1974; Toft, 1985; Walter, 1991) via qualitative, temporal, or spatial differences in how these resources are exploited (Brown & Wilson, 1956; Murray & Brown, 1993; Pfennig, Rice, & Martin, 2006).

Morphological‐related differences in foraging strategies have also been linked to resource partitioning. Multiple examples exist across a broad taxonomic range, including (but not limited to) ungulates (Cromsigt & Olff, 2006; Jarman & Sinclair, 1979; Owen‐Smith, 1985), insects (Takahashi, Tuno, & Kagaya, 2005), reptiles (Losos, Glor, Kolbe, & Nicholson, 2006) and fish (Ross, 1986). These differences in morphology between related species can result in important differences in physiological function and performance (Schmidt‐Nielsen, 1984), and behavior (Dial, Greene, & Irschick, 2008; Peters, 1986) that affect their capacity to escape predators or catch prey (Domenici, 2000; Howland, 1974; Huey & Hertz, 1984). For example, while smaller animals may have faster acceleration (Garland, 1984; Iriarte‐Díaz, 2002; Jackson & Dial, 2011) and greater manoeuvrability (Domenici, 2000; McGuire & Dudley, 2005) than larger relatives, larger animals generally exhibit greater speed (Alerstam, Hedenstrom, & Akesson, 2003; Hedenstrom, 1993; Huey & Hertz, 1984), reduced mass‐specific metabolic rates (Peters, 1986), lower costs of transport, and greater energy stores (Schmidt‐Nielsen, 1972; Tucker, 1970). However, large body size also requires greater average prey intake rates during foraging bouts, potentially leading to selection for an efficient foraging strategy where large amounts of prey can be captured and processed during the short periods of time they are available (Brodie, 1975; Croll, Acevedo‐Gutiérrez, Tershy, & Urbán‐Ramírez, 2001; Goldbogen et al., 2012; Hazen, Friedlaender, & Goldbogen, 2015).

Balaenopterid whales (i.e., rorquals: Balaenopteridae) are the largest animals on earth, and the blue whale (*Balaenoptera musculus*) is the largest animal that ever existed (Nishiwaki, 1950; Werth, 2000). These Balaenopterid whales are characterized by a unique combination of morphological traits (ventral pleats and baleens) allowing them to use lunge feeding to capture and swallow large quantities of small individual prey in a single batch feeding event (Goldbogen et al., 2012; Kawamura, 1980). The availability of large, dense prey aggregations are therefore requisite for successful feeding (Croll et al., 2001; Goldbogen et al., 2011, 2012; Santora, Reiss, Loeb, & Veit, 2010). Such prey aggregations occur in discrete regions of exceptionally high productivity, often associated with fronts, upwelling centers, and steep topography leading to strong spatial and temporal patchiness (Brentnall, Richards, Brindley, & Murphy, 2003; Croll et al., 2005; Santora, Sydeman, Schroeder, Wells, & Field, 2011). This often results in seasonal sympatry of multiple filter‐feeding whale species in spatially‐restricted productive areas. In these instances, resource partitioning facilitates reduced interspecific competition and increased energy gain across species. For example, differences in resource utilization have been described in sympatric humpback (*Megaptera novaeangliae*) and Antarctic minke (*Baleanoptera bonaerensis*) whales around the Western Antarctic Peninsula, where minke whales appear to target deeper krill aggregations than larger humpback whales, as well as exhibit differences in their horizontal spatial distributions (Friedlaender, Johnston, Fraser, Burns, & Costa, 2011; Friedlaender, Lawson, & Halpin, 2009). Little is known, however, on the multiple mechanisms for resource partitioning that may occur simultaneously, such as differences in target prey species, temporal distribution, and spatial distribution in foraging.

Here, we evaluate the hypothesis that multiple differences in foraging behavior facilitate resource partitioning by examining the foraging ecology of two closely‐related sympatric whale species. We integrate complementary datasets, including field surveys, stable isotope analysis, and remote‐sensing of oceanographic variables, to simultaneously evaluate the qualitative (trophic), temporal and spatial differences in foraging of blue and humpback whales. These species co‐occur seasonally in the highly productive waters of Monterey Bay in central California, USA. Understanding the mechanisms for species coexistence is both fundamental to maintaining biodiverse ecosystems and provides insight into the evolutionary drivers of behavioral, morphological and physiological differences in closely related species.

## 2 MATERIALS AND METHODS

### 2.1 Study area

**Figure 1:**
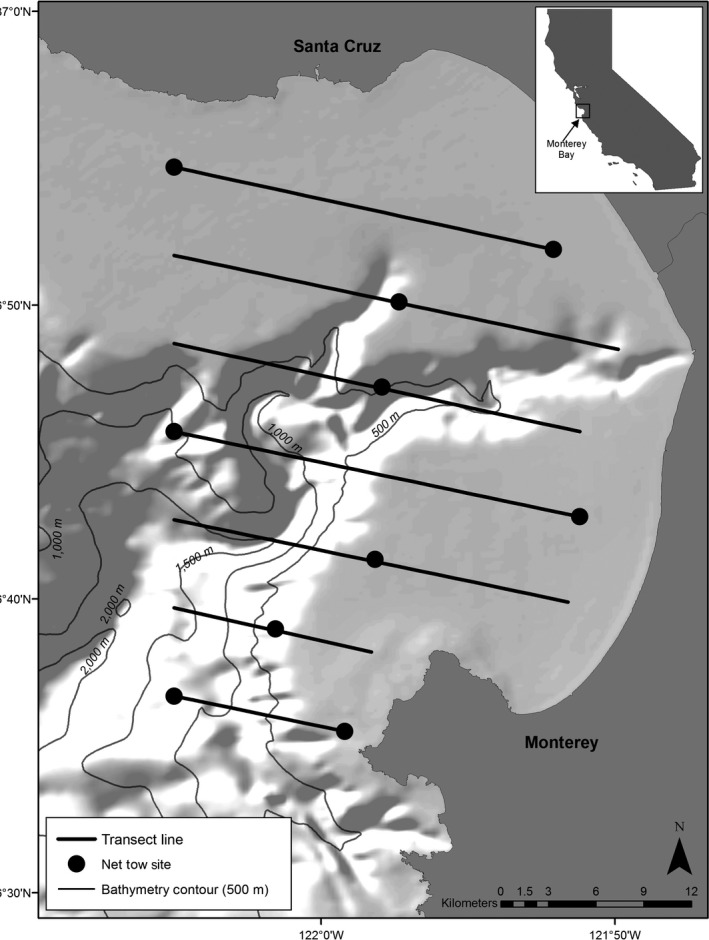
Monterey Bay study area. Monterey Submarine Canyon is characterized by waters deeper than 500 m (light grey lines). Black lines indicate transect lines (10–22 km in length; 5.5 km apart) followed during shipboard surveys to sample krill and forage fish distributions and densities using hydroacoustics, as well as whale distribution and density. Black circles indicate net tow sites to sample zooplankton abundance.

Monterey Bay (36.80°N, 121.90°W) is a large (~1200 km^2^) open bay located off the central California coast (Benson, Croll, Marinovic, Chavez, & Harvey, 2002; Croll et al., 2005; Figure 1) and is divided by the Monterey Submarine Canyon; one of the largest in the world (Shepard & Marshall, 1973). Two nearly equal shallower shelves (up to 140 m deep and 10 to 15 km wide) surround the deeper waters of the canyon located in the center of the bay (Greene, Maher, & Paull, 2002). This highly productive coastal area is strongly influenced by two seasonal upwelling modes: a spring/summer wind‐driven upwelling period and a winter relaxation or downwelling period (Black, Schroeder, Sydeman, Bograd, & Lawson, 2010; Black et al., 2011; Bograd et al., 2009; Schroeder et al., 2009).

### 2.2 Oceanographic data

Satellite oceanographic data hypothesized to influence whale habitat use and previously shown to affect other marine predators were obtained for a 3 × 3 pixel (i.e. 9 km^2^) region centred in Monterey Bay (36.80°N, 121.90°W) for the study period 1997–2006 (Black et al., 2010, 2011; Schroeder et al., 2013). A monthly upwelling index (UI; m^3^ s^−1^ 100 m^−1^) was derived by the NOAA Fisheries, Environmental Research Division (http://www.pfeg.noaa.gov). The index is based on estimates of offshore Ekman transport driven by geostrophic wind stress derived from 6‐hourly, synoptic, surface atmospheric pressure fields (Bakun & Nelson, 1991). Ocean color (Sea‐Viewing Wide Field Sensor, SeaWiFS, and Moderate Resolution Imaging Spectrometer, MODIS AQUA) data were obtained from the NASA Ocean Biology Processing Group (OBPG). Monthly averages of Chlorophyll‐*a* (mg m^−3^) and depth‐integrated primary production (mg C m^−2^ day^−1^) were derived using data processed with the standard OBPG methods for chlorophyll‐*a*, and the standard Vertically Generalized Production Model (VGPM) for primary production as described by Behrenfeld and Falkowski (1997), modified to use a non‐varying chlorophyll‐specific primary productivity term (P^B^_opt_) as described by Kudela, Cochlan, Peterson, and Trick (2006). A cumulative upwelling index (CUI) at 36°N, 122°W and the North Pacific High (NPH) position and strength indices (NPH's monthly areal extent (A) and maximum pressure (Pmax)) were included to examine broader scale forcing of the Monterey Bay Ecosystem (Schroeder et al., 2013).

### 2.3 Prey distribution, density, and phenology

#### 2.3.1 Line‐transect surveys

Shipboard line‐transect surveys to sample krill and forage fish distributions and densities were conducted monthly from May to November 1997 to 2006. Additional surveys were conducted in January 2003 to 2006 and in March 2003 to 2005. Each survey consisted of seven transect lines ranging from 10 to 22 km in length and spaced 5.5 km apart (Figure 1). A survey totaled ~126 km and was typically completed in two consecutive days at a ship speed of 18.5 km h^−1^ (10 knots). The entire survey area, ~909 km^2^, included all of Monterey Bay and the waters off the Monterey Peninsula (except nearshore regions) beginning at the 55 m (30 fathom) isobaths and extending WNW to 122.08°W longitude (Figure 1, for more details on the survey design, see Benson et al., 2002; Croll et al., 2005).

#### 2.3.2 Hydroacoustic survey and net sampling

Krill (i.e., euphausiids) and forage fish densities were estimated along survey transects from 1997 to 2006 and from 2003 to 2006, respectively, using Simrad echosounders. Krill hydroacoustic data were analysed based on the methods described in Croll et al. (1998) and Hewitt and Demer (1993) to provide relative integrated measures of acoustic backscatter for euphausiids for the whole survey grid. Krill schools were identified and scrutinized from other scattering organisms based upon school morphology and frequency‐specific differences in backscatter strength. Volume backscattering was integrated vertically from 5 m below the surface down to either 200 m or 5 m above the bottom, and averaged over 1 km horizontal intervals of each transect. Krill schools detected hydroacoustically were additionally confirmed by periodic targeted net tows. The net was towed obliquely to either 10 m above the bottom or to 200 m depth. Krill density (number/1000 m^3^) and zooplankton biovolume (total zooplankton displacement volume, mL/1000 m^3^) were calculated based on conventional MOCNESS/BONGO net sampling as described in Marinovic, Croll, Gong, Benson, and Chavez (2002).

Forage fish schools were identified from hydroacoustic records based upon school morphology and differential target strengths, as organisms with swim bladders (e.g., many fishes) scatter greater at lower frequencies than those without (e.g., krill; Simmonds & MacLennan, 2005). Due to difficulties associated with distinguishing among fish species using hydroacoustic data, we included all hydroacoustic records matching our schooling fish criteria under the general term forage fish. Forage fish density was calculated as school encounter rate, where individual schools were identified and the number of schools encountered per kilometer of transect‐line was calculated.

#### 2.3.3 Spatio‐temporal patterns

To examine temporal patterns in prey distribution and density, hydroacoustic and net sampling data were averaged monthly across all sampled stations. These monthly averages were then combined into a time series (1997–2006 for krill and 2003–2006 for fish) to generate long‐term average patterns for Monterey Bay. To examine spatial patterns in prey distribution and density, and in particular the importance of the Monterey Submarine Canyon in structuring spatial heterogeneity of critical prey, the proportion of krill schools or forage fish schools north and south of the Monterey Submarine axis (36.81°N) was calculated for each survey from 2003 to 2006. Finally, the densities of krill or forage fish were estimated for prey encountered in waters <105 m (i.e. on the continental shelf) or ≥105 m depth (i.e. off the continental shelf) for each survey from 2003 to 2006 and averaged by month.

### 2.4 Whale distribution, density, and phenology

#### 2.4.1 Line‐transect surveys and methods

Surveys were conducted during 2 consecutive days each month from May through November 1997 to 2006, using standard line‐transect methods for marine mammals developed by the US National Marine Fisheries Service (Benson et al., 2002). Additional surveys were completed during 2 consecutive days in January and March 2003 to 2006. In 2007, surveys were conducted 1 day a month (5 transect lines totaling 82 km) during January, March, May, July, August, and November (Figure 1; for more details on the survey design, see above and Benson et al., 2002; Croll et al., 2005).

Sighting information (time, latitude, longitude, species, number of individuals, sighting cue, method of detection, compass bearing, and number of reticle marks down from the horizon) and weather conditions were recorded. Whales were identified to the lowest possible taxonomic level, i.e. humpback whale *Megaptera novaeangliae* or blue whale *Balaenoptera musculus*. Whale density estimates were calculated using standard marine mammal line transect methods (Barlow et al., 1995; Buckland, Anderson, Burnham, & Laake, 1993). Sighting distances of whale groups to the transect line were calculated from the compass bearings and reticle readings. Monthly whale densities (number of individuals per km^2^) were calculated from line transect data using the Multiple Covariate Distance Sampling (MCDS) analysis engine in Distance software (Buckland, Anderson, Burnham, & Laake, 2005; Thomas et al., 2010). Detailed description of density analysis is presented in Burrows, Harvey, Newton, Croll, and Benson (2012).

#### 2.4.2 Whale watching dataset

To complement the line‐transect surveys, opportunistic sightings of blue and humpback whales were obtained between 1993 and 2004 from Monterey Bay Whale Watch Company. Because effort per trip was not recorded and the number of trips per month varied between months and years from a minimum of one trip to a maximum of 26 trips per month, opportunistic data only provide relative estimates of whale presence in the bay. During each whale‐watching trip, sighting information (time, latitude and longitude or approximate position relative to the coast, species, number of individuals) were recorded. Total number of sightings per day per species was computed to estimate relative daily abundance (calculated as mean number of individual whale sightings per day per species). While opportunistic data are not appropriate to obtain reliable absolute density estimates, they can be used to examine phenology, or the timing of when both species used the bay, and seasonal trends. To examine species phenology, we only used years where trips were conducted from at least April to November (*n* = 6 years, 1996–1998; 2000–2002), encompassing the main season for both species. We used two criteria to define the arrival date: (i) arrival date is the date of first sighting of a species, (ii) this first sighting should be followed by regular sightings over the following 10 days. The departure date was considered as the date of last sighting of a species in the bay.

#### 2.4.3 Spatio‐temporal patterns

To examine temporal patterns in whale distribution, density, and relative abundance, survey data were averaged monthly across all sampled stations and whale watching data were averaged monthly across all trips. These monthly averages were then combined into a time series (1997–2007 for line‐transect data, 1993–2004 for whale‐watching data) to generate long‐term average patterns for Monterey Bay. The proportion of sightings in waters <105 m (i.e. on the continental shelf) or ≥105 m depth (i.e. off the continental shelf) was calculated for each year from 1997 to 2007 to examine the importance of the shelf break in structuring spatial heterogeneity of whale species. The importance of the Monterey Submarine Canyon in structuring spatial heterogeneity of whale species was investigated by calculating the proportion of humpback and blue whales sightings north and south of the Monterey Submarine axis (36.81°N) over the same time period.

### 2.5 Whale trophic position and inferred diet

To examine trophic position of blue and humpback whales, we conducted stable isotope analysis on skin samples obtained from biopsy darts. Stable isotope analysis is a powerful tool for assessing the diet composition and trophic level of predators (Newsome, del Rio, Bearhop, & Phillips, 2007; Post, 2002). All samples used in this study were obtained in 2005 as part of a larger biopsy sampling program (Fleming, Clark, Calambokidis, & Barlow, 2015). A total of 39 individuals were sampled (25 humpback, 14 blue whale) along the U.S. California Current range of the whales (Washington to California). Skin samples were collected using a small stainless steel biopsy dart fired from a crossbow (summarized in Ralls and Hoelzel (1992)). Cetacean skin is a metabolically active tissue, which reflects recent dietary inputs, with a mean isotopic incorporation rate of 163 ± 91 days (Busquets‐Vass et al., 2017). Each dart was fitted with a flange that regulated the penetration of the dart and caused recoil after sampling to release the dart from the skin. Darts were collected and the sampled tissue was frozen or stored in dimethyl sulfoxide (DMSO) or ethanol. A recent study on blue whales found no difference in isotopic values extracted from skin samples for different preservation methods (Busquets‐Vass et al., 2017).

Samples were separated by species and early vs. late upwelling/oceanic time period in Monterey Bay, using July 15th as the approximate transition date (Pennington & Chavez, 2000). Tissue samples were sent to the Colorado Plateau Stable Isotope Library, Northern Arizona University (Flagstaff, Arizona). Samples were oven dried followed by lipid extraction (Soxhlet) and homogenized for determination of δ^15^N. Nitrogren isotope ratios are useful for assessing trophic position, as higher trophic levels have higher values of δ^15^N (Post, 2002). In addition, δ^15^N in humpback whales have been shown to be significantly positively related to forage fish abundance and negatively related to krill abundance in the California Current System, allowing diet inferences (Fleming et al., 2015). Analyses were conducted using a Finnigan Delta Plus isotopic ratio mass spectrometer (Thermo Electron Corporation, Waltham, MA). Detailed description of methods is presented in Fleming et al. (2015).

All statistical analyses were conducted in R 3.2.2 (R Core Team 2016). All results are reported as mean ± *SD*, unless otherwise noted.

## 3 RESULTS

### 3.1 Oceanographic conditions and predator/prey temporal distribution

**Figure 2:**
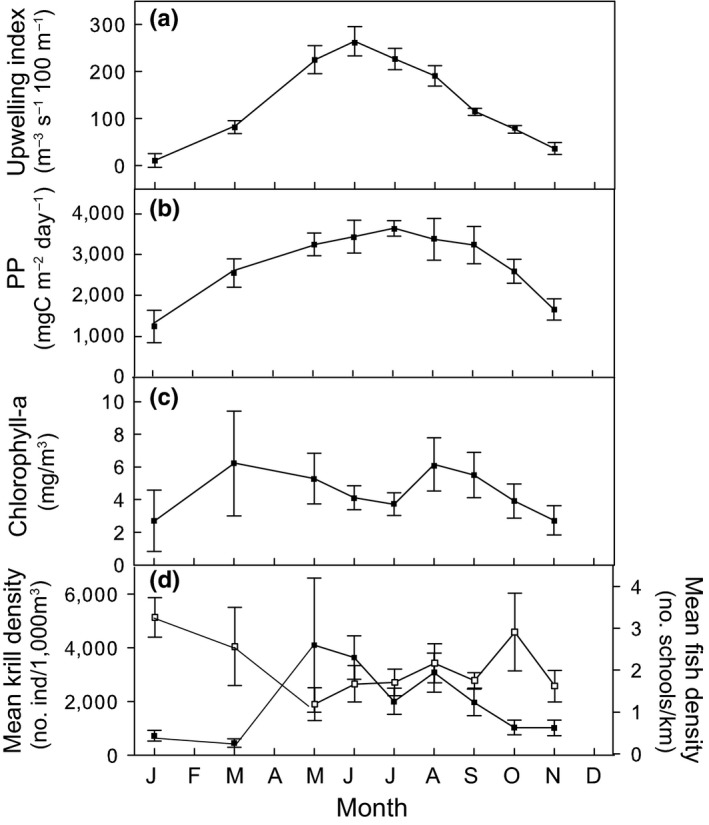
Monthly mean (±1 *SE*; a) upwelling index, (b) primary production, (c) Chlorophyll a, (d) krill density from 1997 to 2006 (black dots) and forage fish density from 2003 to 2006 (white dots) in Monterey Bay, California

Monterey Bay is characterized by strong seasonal upwelling beginning in February–March, peaking in June (264.0 ± 30.8 m^−3^ s^−1^ 100 m^−1^) and diminishing around August–September (Figure 2a, see also (Pennington & Chavez, 2000). During this period, strong northwest winds cause the upwelling of cool, deep, nutrient‐rich waters to the surface, which support increased primary and secondary production. Mean primary productivity is highest between June and August (>3300 mg C m^−2^ day^−1^; Figure 2b) whereas Chlorophyll‐*a* peaks first in March (6.2 ± 3.2 mg m^−3^) and again in August (6.1 ± 1.6 mg m^−3^; Figure 2c).

These oceanographic conditions are mirrored by bimodal peaks in krill May (5.9 ± 0.7 month; 22.7 ± 42.1 ind 1000 m^−3^) and August (8.7 ± 0.7 month; 17.1 ± 12.3 ind 1000 m^−3^), and in forage fish density in January (1.5 ± 0.9 month; 17.3 ± 10.5 schools km^−1^) and October (10.0 ± 0.4 month; 15.4 ± 20.6 schools km^−1^), respectively (Figure 2d). During the peak in krill density in May, fish density was at its lowest (6.3 ± 8.7 schools km^−1^ Figure 2d).

**Figure 3:**
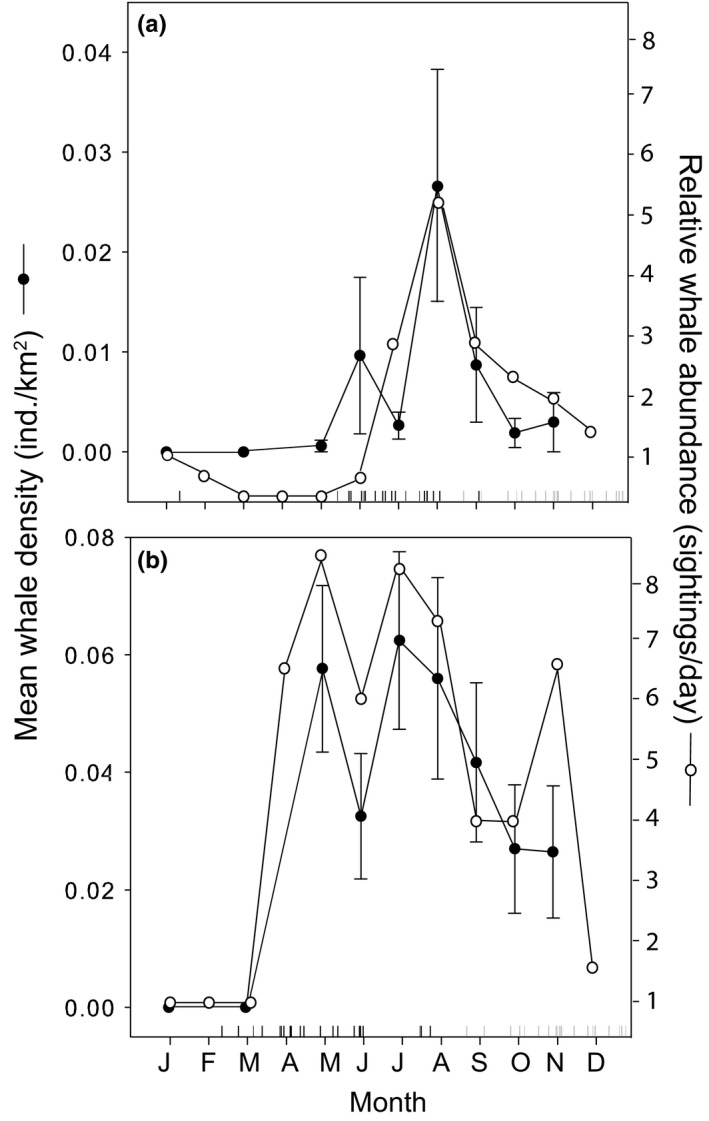
Monthly mean (±1 *SE*) whale density from 1997 to 2007 from line‐transect surveys (black dots) and monthly relative whale abundance (calculated as mean number of individual whale sightings per day per species, see Appendix 1 for mean and *SE* values) from 1993 to 2004 from Whale Watch daily sightings data (white dots) in Monterey Bay, California. (a) Blue whale, (b) humpback whale. In each panel, black and grey vertical marks along the *x* –axis show the dates of first and last regular sightings of blue (a) and humpback (b) whales in Monterey Bay each year from 1993 to 2004.

From whale watch records between 1993 and 2004, mean blue whale arrival and departure dates to Monterey Bay were July 22th (±22 days) and October 31st (±49 days), respectively (Figure 3a). During the same period mean humpback whale arrival and departure dates to Monterey Bay were May 12th (±37 days) and December 8th (±14 days), respectively (Figure 3b). Accordingly, blue whales had a more peaked seasonality (kurtosis = 3.2; leptokurtic distribution) than humpback whales (kurtosis = −1.3; platykurtic distribution; Figure 3).

Relative monthly abundance, measured as the mean number of sightings per day in a given month, of blue whales peaked in August (8.7 ± 1.5 month, Figure 3a), whereas humpback whale relative abundance was bimodal with peaks occurring in late April (4.8 ± 0.4 month) and July (7.6 ± 1.4 month, Figure 3b). Relative blue whale monthly abundance was positively correlated with the monthly cumulative upwelling index (CUI; Table 1). Relative humpback whale monthly abundance was positively correlated with the monthly upwelling index at 36N, monthly krill density, and monthly areal extent (A) and maximum pressure (pmax) of the NPH (Table 1). Annual peak in the relative abundance of blue whales was not correlated with the density of krill in July or August, however annual peak in the relative abundance of humpback whales was positively correlated with the density of krill in June (Spearman's correlation, ρ = 0.88, *p* = .07, *n* = 5).

**Table 1:** Spearman's correlation results between monthly mean whale (blue and humpback) density and relative abundance and monthly mean biophysical factors in Monterey Bay, California

	Blue whale		Humpback whale	
	Monthly density	Monthly abundance	Monthly density	Monthly abundance
UI_36N (m^−3^ s^−1^ 100 m^−1^)	**ρ = 0.25, ** ***n*** ** = 83, ** ***p*** ** = .022**	ρ** = **0.09, *n* = 173, *p* = .22	**ρ = 0.30, ** ***n*** ** = 83, ** ***p*** ** = .007**	**ρ = 0.47, ** ***n*** ** = 173, ** ***p*** ** = .00003**
CUI (m^−3^ s^−1^ 100 m^−1^)	0.19, 83, 0.08	**0.49, 173, 0.00002**	0.06, 83, 0.60	−0.08, 173, 0.49
NPH_area	0.02, 83, 0.86	−0.09, 173, 0.43	**0.34, 83, 0.002**	**0.36, 173, 0.002**
NPH_PMax	0.02, 83, 0.87	−0.03, 173, 0.79	**0.33, 83, 0.002**	**0.36, 173, 0.002**
PP (mg C m^−2^ day^−1^)	0.02, 72, 0.87	−0.02, 42, 0.92	0.13, 72, 0.27	0.28, 42, 0.08
Chl_a (mg m^−3^)	−0.06, 72, 0.63	0.07, 42, 0.65	0.03, 72, 0.77	0.25, 42, 0.13
Fish density (school km^−1^)	0.05, 36, 0.78	0.14, 8, 0.76	−0.13, 35, 0.44	−0.35, 8, 0.55
Krill density (no. ind 1000 m^−3^)	0.13, 72, 0.25	−0.17, 45, 0.29	**0.31, 75, 0.006**	**0.41, 45, 0.006**
Krill school density (school km^−1^)	**0.49, 36, 0.003**	−0.29, 8, 0.53	0.05, 35, 0.76	−0.61, 8, 0.26

UI_36N, upwelling index at 36°N; CUI, cumulative upwelling index; NPH_area, North Pacific High areal extent; NPH_PMax, North Pacific High maximum pressure; PP, primary productivity; Chl_a, chlorophyll‐*a* concentration. Results in bold indicate a significant relationship (*p* < .05). *n*, number of monthly values.

Mean blue whale density began increasing in May, peaked on average mid‐July (7.5 ± 0.7 month; 0.027 ± 0.015 ind km^−2^) and decreased until November (Figure 3a). Mean humpback whale density peaked in late May (mean ± *SD* = 5.7 ± 0.9 month; 0.057 ± 0.014 ind km^−2^) and late August (mean ± *SD* = 8.7 ± 1.3 month; 0.062 ± 0.015 ind km^−2^) and remained relatively high until November (>0.026 ind km^−2^, Figure 3b). Annual peak density of blue whales was positively correlated with the maximum biovolume of zooplankton (Spearman's correlation, *s* = 55.7, ρ = 0.66, *p* = .004, *n* = 11). Annual peak density of humpback whales was positively correlated with the maximum yearly value of the upwelling index at 36N (Pearson's correlation, *t*_9_ = 2.56, *r* = 0.65, *p* = .03). Blue whale monthly density was significantly correlated with the monthly upwelling index at 36N and the monthly krill school density (Table 1). Humpback whale monthly density was positively correlated with the monthly upwelling index at 36N, monthly areal extent (area) and maximum pressure (pmax) of the NPH (Schroeder et al., 2013) and monthly krill density (Table 1).

### 3.2 Predator/prey spatial distribution

**Figure 4:**
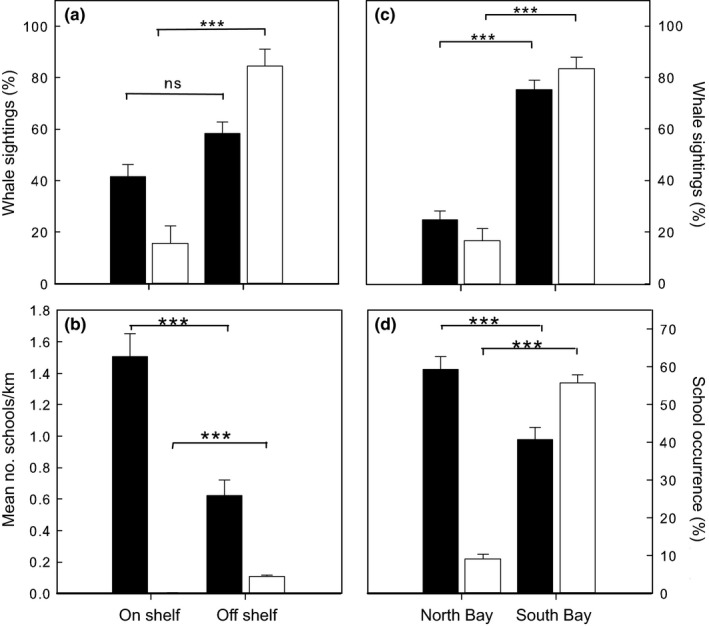
Spatial distribution of whales, krill and fish schools in Monterey Bay California. (a) Mean percentage (±1 *SE*) of humpback (black bars) and blue (white bars) whale sightings on and off the continental shelf in Monterey Bay. (b) Mean density (±1 *SE*) of fish (black bars) and krill (white bars) schools on and off the continental shelf in Monterey Bay. (c) Mean percentage (±1 *SE*) of humpback (black bars) and blue (white bars) whale sightings in north (>36.81°N) and south (<36.81°N) Monterey Bay. (d) Mean percentage (±1 *SE*) of fish (black bars) and krill (white bars) schools in north (>36.81°N) and south (<36.81°N) Monterey Bay. ns: not significant, ***p < .01, Mann–Whitney Test

Over a ten‐year period, sightings of blue whales were more common off the continental shelf than on the continental shelf (84.4 ± 6.8% vs. 15.6 ± 6.8%; Mann–Whitney Test: *W* = 125.5, *p* < .001, *n* = 18 yearly values, no blue whale sightings were recorded in 2006 and 2007, Figure 4a). In contrast, there was no significant difference in the number of humpback whale sightings on vs. off the continental shelf (58.3 ± 4.5% vs. 41.7 ± 4.5%; Mann–Whitney Test: *W* = 156.5.0, *p* = .053, *n* = 22 yearly values, Figure 4a). The mean density of forage fish schools was nearly double on shelf than off shelf (1.5 ± 0.15 schools km^−1^ vs. 0.6 ± 0.09 schools km^−1^; Mann–Whitney Test: *W* = 1646.0, *p* < .0001, *n* = 70 monthly values, Figure 4b) whereas the opposite pattern was observed for krill schools, which were 28 times less abundant on shelf than off shelf (0.004 ± 0.002 schools km^−1^ vs. 0.11 ± 0.01 schools km^−1^, Mann–Whitney Test: *W* = 632.0, *p* < .0001, *n* = 70 monthly values, Figure 4b).

In addition, there were significantly more sightings of both blue and humpback whales south of Monterey Bay Canyon than north (Blue whale: 83.3 ± 19.9% vs. 16.7 ± 19.9%, Mann–Whitney Test: *W* = 125.5, *p* < .001, *n* = 18 yearly values; Humpback whale: 75.4 ± 16.7% vs. 24.6 ± 16.7%, Mann–Whitney Test: *W* = 184.0, *p* < .001, *n* = 22 yearly values, Figure 4c). Similarly, the proportion of krill schools was significantly greater south of Monterey Bay Canyon than north (55.6 ± 2.7% vs. 9.0 ± 1.4%, Mann–Whitney Test: *W* = 638.0, *p* < .0001, *n* = 70 monthly values, Figure 4d). In contrast, the proportion of fish schools was significantly lower south of Monterey Bay Canyon than north (40.8 ± 3.1% vs. 59.2 ± 3.4%, Mann–Whitney Test: *W* = 1547.0, *p* < .001, *n* = 70 monthly values, Figure 4d).

### 3.3 Inferred predator diet

**Figure 5:**
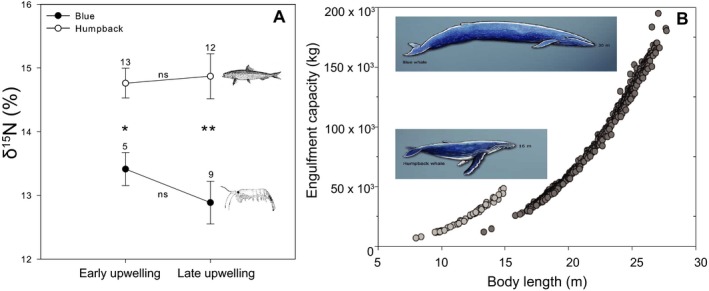
Humpback and blue whale inferred diet and engulfment capacity. (a) Mean isotope values (±1 *SE*) for humpback whales (white circles) and blue whales (black circles) before and after 15th July 2015 (transition between early and late upwelling periods) in Monterey Bay, California. ns: not significant, *p < .05, **p < .01, Mann–Whitney Test. Lower δ^15^N values may represent a diet dominated by krill, higher δ^15^N values may represent a diet dominated by forage fish. (b) Relationship between engulfment capacity and body length in the humpback whale (white dots) and blue whale (grey dots) based on a mechanistic model from Goldbogen et al. (2011).

Mean δ^15^N values were significantly greater in humpback whales than blue whales (before July 15th: 14.76 ± 0.81 vs. 13.41 ± 0.52; after July 15th: 14.87 ± 1.16 vs. 12.89 ± 0.95; Mann–Whitney test, *P* < .05 in all cases, Figure 5a) suggesting that in 2005, when biopsy samples were collected, humpback whales likely fed on higher‐trophic level prey, e.g. forage fish, compared to blue whales. For both species, mean δ^15^N values were not significantly different before versus after July 15th 2005 (Figure 5a), indicating no significant seasonal shifts.

## 4 DISCUSSION

Long‐term studies integrating complementary datasets offer unique opportunities to understand the ecology of wide‐ranging and long‐lived species. Our study highlights the differences in the spatio‐temporal distribution and foraging behavior of two seasonally sympatric whale species (blue and humpback), and suggests that these distributional and behavioral differences facilitate reduced competition between these two closely related species.

### 4.1 A highly productive hotspot

Monterey Bay has been described as a highly productive “hotspot” supporting high concentrations of forage fishes (e.g., anchovy, sardines), krill and top predators (Santora et al., 2012). Our long‐term dataset ranging from physical oceanography to top predator ecology showcases this productivity and underscores its seasonal nature. Our study shows that the spring transition to summertime upwelling leads to a peak in phytoplankton standing crop in March–April which results in krill density peaking in May/June (see also Croll et al., 2005; Marinovic et al., 2002). A secondary peak in krill density occurs again in August as individual krill spawned during late upwelling enter the population, and adults, resulting from the primary recruitment pulse in May, are still surviving in the population (see also Marinovic et al., 2002; Croll et al., 2005). Thus, while krill are present year‐round in Monterey Bay, their density is strongly seasonal, with two peaks largely driven by physical/biological coupling of upwelling dynamics. Our study also reveals that forage fishes were likewise encountered throughout the year, but similarly displayed strong seasonality associated with upwelling dynamics (Black et al., 2011). However, in an opposite pattern to krill density, forage fish density was greatest during relaxation periods.

Our results show that krill species in Monterey Bay are associated with the Monterey Submarine Canyon shelf break, and are more abundant in the southern part of the bay and off the continental shelf compared to on the shelf. The relationship between krill density and steep topographic features – particularly channels and shelf edges – has been previously described in Monterey Bay (Croll et al., 1998, 2005; Santora et al., 2011) and elsewhere (Lavoie, Simard, & Saucier, 2000). In contrast, forage fishes were more abundant on the continental shelf and north of the Monterey Bay Canyon. Upwelling dynamics lead to temporal and spatial lags between peak upwelling, peak primary production, and peak secondary production and enhanced grazing (Wilkerson, Lassiter, Dugdale, Marchi, & Hogue, 2006). These temporal and spatial lags, combined with the upwelling shadow mechanism described for Monterey Bay (Graham & Largier, 1997), result in enhanced zooplankton prey abundance downstream from upwelling center and within the upwelling shadow region of northern Monterey Bay where forage fish appear to concentrate.

The timing of seasonal upwelling is therefore a primary factor in determining temporal patterns of prey aggregations in the Bay, whereas spatial patterns of prey are strongly influenced by the interaction of seasonal upwelling with the local geomorphology of the continental shelf break and the submarine canyon. These spatio‐temporal differences in prey distribution and density, in turn, are closely linked with the movements and foraging ecology of both humpback and blue whales.

### 4.2 Foraging strategies and resource partitioning

Blue and humpback whales are both filter feeders with high energetic demands, requiring dense prey aggregations to feed successfully (Croll et al., 2001; Goldbogen et al., 2011). Blue whales are known to exclusively feed on krill (Sears & Perrin, 2009), whereas humpback whales have a more diverse diet, including krill as well as small schooling fish (Mann, 2000). Such dense aggregations only occur in discrete regions of exceptionally high productivity and are characterized by strong spatial and temporal patchiness, presenting the potential for resource competition between these species.

Our results suggest that due to their more generalist diet, humpback whales are able to switch prey depending on relative abundances, which may serve as a mechanism to reduce interspecific competition. Over the study period, humpback relative abundance and density were consistently high throughout the entire upwelling season; even during months when forage fish density was low, i.e., in May and June. The annual peak in the relative abundance of humpback whales was positively correlated with the density of krill in June, suggesting that humpbacks may target the early season peak in krill density. Later in the season, we suggest that humpbacks switch to feeding upon forage fishes as fish density increases. Our stable isotope analysis for 2005 supports this hypothesis: humpbacks had greater mean δ^15^N values than blue whales, suggesting a diet comprised of higher trophic level prey. While we could not verify δ^15^N values of krill and forage fish species at the time of sampling, stable isotopes processed using the same methods in humpback whales in the California Current accurately reflected changes in prey choice and availability between krill and forage fish over multiple years (Fleming et al., 2015). In addition, a review of the literature for isotopic values of prey (i.e. krill and forage fish species) in the California Current System (Becker, Peery, & Beissinger, 2007; Brodeur, Suchman, Reese, Miller, & Daly, 2008; Miller et al., 2013; Sydeman, Hobson, Pyle, & McLaren, 1997) suggested δ^15^N values for krill are on average 2 to 3% lower than δ^15^N values for forage fish species, which is agreement with our interpretation of the results. In 2005, competition for krill in the California Current may have been particularly elevated, as conditions in Monterey Bay were anomalously warm (Jahncke et al., 2008; Kudela et al., 2006), which is associated with low zooplankton productivity (Mangel, Marinovic, Pomeroy, & Croll, 2002). The abundance of krill in 2005 was the lowest recorded during the 11 year‐period of our study. A longitudinal study of humpback whale isotope analysis between 1993 and 2012 found a significant shift toward higher δ^15^N values in 2005, suggesting that the species may have responded to decreased availability of krill that year by preferentially targeting forage fishes (Fleming et al., 2015).

In comparison, blue whales had a narrower peaked abundance distribution than humpback whales. Humpback whales often remain for a long period of time (~6 months) in a single foraging area (Baker et al. 2013), whereas blue whales have been shown to move great distances between regions of seasonal productivity (Calambokidis, Barlow, Ford, Chandler, & Douglas, 2009), feeding in a more localized area for about 3 weeks before migrating to another area (Bailey et al., 2009; Irvine, Mate, Winsor, & Palacios, 2014). Humpback whale relative abundance, density and time of arrival in Monterey Bay were linked with broad scale oceanographic parameters: the intensity of summer upwelling in the California Current System and/or the amplitude of the North Pacific High. Both have been previously shown to influence other biological processes in the California Current System, such as seabird egg laying date and fledging success (Black et al., 2010, 2011; Schroeder et al., 2013). While humpbacks seemed to respond more to large scale forcing, blue whales seemed to be influenced by more localised cues. The annual peak in blue whale density which occurred on average between mid‐July and August, at the same time as the secondary, more predictable, late summer peak in krill, was correlated with the maximum biovolume of zooplankton in the Bay. Our results also reveal an interesting difference between both species: while monthly humpback whale density was linked with the monthly krill density in Monterey Bay, monthly blue whale density was more closely correlated with the monthly density of krill schools. Blue whales have extremely high prey demand, requiring up to two tons of prey per day (Goldbogen et al., 2011; Rice, 1978). This constrains them to feed upon extremely dense euphausiid schools, presumably leading to a relatively high threshold density of both prey and schools for feeding events to be profitable (Acevedo‐Gutierrez, Croll, & Tershy, 2002; Goldbogen et al., 2011; Hazen et al., 2015). This may be why blue whales in Monterey Bay were attuned to predictable and low variance peaks in prey compared to periods of higher and more variable prey density and timed their arrival with the late summer peak in krill.

Within the bay, the two species overlapped spatially; both species were preferentially found south of the Monterey Canyon. However, blue whales were mainly observed offshore, where krill abundance was the highest, whereas humpback whales foraged both on and off the continental shelf, most likely related to their target prey. Blue whales therefore appear to target specific spatial and temporal patterns within Monterey Bay to efficiently feed on predictably dense krill schools along the shelf break and off‐shelf (Croll et al., 2005). Moreover, blue whales exploit a much broader foraging area than humpback whales during the summer, foraging from British Columbia to California (Bailey et al., 2009; Burtenshaw et al., 2004; Irvine et al., 2014; Mate, Lagerquist, & Calambokidis, 1999). Humpback whales tend to utilize more spatially confined feeding areas as demonstrated by strong fidelity to specific feeding regions based on both mtDNA (Baker et al., 2013) and photo‐identification (Calambokidis et al., 1996, 2001). Similar examples of larger species foraging at broader scales than their smaller‐bodied congeners exist in terrestrial species, such as ungulates (Laca, Sokolow, Galli, & Cangiano, 2010; Ofstad, Herfindal, Solberg, & Saether, 2016).

Our study suggests that sympatric blue and humpback whales use distinct foraging strategies during the upwelling season in the waters of Monterey Bay. This likely facilitates sympatry by decreasing competition for their primary prey, krill. A number of marine species feeding on a common resource have similarly been shown to partition resources through a combination of differing prey selection, habitat utilization, timing of foraging, and foraging efficiency, including sympatric seabirds (Cherel et al., 2008; Gonzalez‐Solis, Croxall, & Wood, 2000; Wilson, 2010), fur seals (Page, McKenzie, & Goldsworthy, 2005), dolphins (Browning, Cockcroft, & Worthy, 2014), and whales (Friedlaender et al., 2009, 2011; Ingram, Walshe, Johnston, & Rogan, 2007; Witteveen, De Robertis, Guo, & Wynne, 2015). Our study demonstrates how species may simultaneously partition resources qualitatively, temporally and spatially, ultimately leading to reduced interspecific competition.

### 4.3 Character displacement hypothesis

We suggest that the differences in habitat use and foraging behavior observed between blue and humpback whales in our study are consistent with interspecific differences in body size and lend support for the character displacement hypothesis. The character displacement hypothesis contends that competition between species drives the evolution of differences in morphology (Brown & Wilson, 1956; Grant, 1972; Grant & Grant, 2006). For example, a study examining the spatial association between baleen whales and their principal prey, Antarctic krill *Euphausia superba,* near the South Shetland Islands (Antarctic Peninsula), found that humpback, fin and minke whales partition foraging habitat based on the size of their prey (Santora et al., 2010): humpback were associated with small (<35 mm) juvenile krill, fin whales *Balaenoptera physalus* were associated with large (>45 mm) mature krill located offshore and Antarctic minke whales were associated with intermediate sized krill (35–44 mm). Different size and morphology of baleen plates may have influenced these whales’ divergent prey selections (Gaskin, 1982).

Differences in overall body size may also manifest in decreased competition, as these lead directly to differences in cost of transport, energy storage, and prey capture rates, and indirectly to differences in fasting and migratory abilities (Dial et al., 2008; Domenici, 2000; Howland, 1974; Huey & Hertz, 1984; Peters, 1986). The extreme body size of blue whales is associated with both a lower mass‐specific metabolic rate and cost of transport, an advantage for long distance travelling (Goldbogen et al., 2011). Large lipid reserves can also serve as a buffer from variability in coastal productivity, which is particularly important for a predator specialised in a patchy and ephemeral resource such as krill. The higher mass‐specific engulfment capacity of blue whales has also been suggested to be more efficient for krill feeding, while the humpback's lower capacity values and higher manoeuvrability related to its smaller body size (Domenici, 2000; McGuire & Dudley, 2005) may be better for exploiting more agile prey like fish (Goldbogen et al., 2011; Figure 5b). Evaluating the extent to which differential selection on body size may be occurring between the two species to facilitate resource partitioning would be a ripe area for future research.

## 5 CONCLUSION

Areas of high seasonal productivity, such as Monterey Bay, are critical foraging areas for a wide range of species (Block et al., 2011). In these areas, the probability of overlapping distributions and resource competition among species is high and can therefore present an opportunity to examine, in the field, how competition for resources may drive resource partitioning between seasonally sympatric, closely related species. Ultimately, such partitioning can lead to behavioral, morphological, and physiological character displacement between sympatric species. Our long‐term study using complementary approaches reveals several mechanisms operating simultaneously that may facilitate coexistence among some of the largest co‐occurring species on Earth.

## AUTHOR CONTRIBUTIONS

SF, SB, EH and DC conceived the study. KMN, JC, JB, JG, JH, BM, BT and DC collected and contributed data. SF performed the analyses with contributions from EH, JG and BA. SF, DC and BA wrote the manuscript, with input from all authors.

## DATA ACCESSIBILITY

We will publicly archive data supporting our findings on Dryad.

## APPENDIX 1

### 1.1

Monthly mean ± 1 *SE* relative whale abundance (calculated as mean number of individual whale sightings per day per species) from 1993 to 2004 from Whale Watch daily sightings data in Monterey Bay, California

**Table:**

Month	Number of humpback whale day^−1^	Number of blue whale day^−1^
January	1.0 ± 1.0	1.0 ± 1.0
February	1.0 ± 1.0	0.5 ± 0.5
March	1.0 ± 0.0	0.0 ± 0.0
April	6.6 ± 1.0	0.0 ± 0.0
May	8.7 ± 0.9	0.0 ± 0.0
June	6.0 ± 1.2	0.3 ± 0.1
July	8.2 ± 0.9	2.8 ± 0.6
August	7.2 ± 0.8	5.3 ± 0.6
September	3.9 ± 0.6	2.8 ± 0.5
October	3.9 ± 1.0	2.3 ± 0.4
November	6.9 ± 1.7	1.9 ± 0.4
December	1.4 ± 0.4	1.4 ± 0.7
